# Sensitive Biomarker Analysis of Xue-Fu-Zhu-Yu Capsule for Patients with Qi Stagnation and Blood Stasis Pattern: A Nested Case-Control Study

**DOI:** 10.1155/2019/7182865

**Published:** 2019-11-18

**Authors:** Guang Chen, Haoqiang He, Kun Hu, Jialiang Gao, Jun Li, Mei Han, Jie Wang

**Affiliations:** ^1^Department of Cardiology, Guang'anmen Hospital, China Academy of Chinese Medical Sciences, Beijing 100053, China; ^2^Graduate School, Beijing University of Chinese Medicine, Beijing 100029, China; ^3^Center for Evidence-based Chinese Medicine, Beijing University of Chinese Medicine, Beijing 100029, China

## Abstract

**Objectives:**

To identify the sensitive biomarker to predict the effectiveness of Xue-Fu-Zhu-Yu capsules (XFZYC).

**Methods:**

This nested case-control study included 5 patients with response to XFZYC in the treatment group, 5 patients with no response to XFZYC also in the treatment group, and 5 patients in the control group treated with placebo who participated in the previous RCT. The mRNAs, miRNAs, lncRNAs, and circRNAs were sequenced by next-generation sequencing and differential-expressed (DE) RNAs were identified if *p* value ≤ 0.05 and fold change ≥2, bioinformatics analysis was conducted in terms of function annotations and signaling pathways, and then sensitive biomarker was analyzed based on real-time PCR.

**Results:**

The distributions of clinical characteristics between the selected participants from treatment group and placebo group were well balanced. A total of 1156 DE RNAs, 388 miRNAs, 1954 lncRNAs, and 560 circRNAs were identified, which was associated the mechanism of XFZYC and composed the targeted potential biomarkers for further real-time PCR. The DE RNAs were enriched in KEGG pathways pertaining to pathogenesis of Qi Stagnation and Blood Stasis- (QS & BS-) & associated diseases such as coronary heart disease and digestive diseases. The expression level of FZD8 was significantly higher in response patients than that in nonresponse patients (*p* = 0.041) and circRNA_13799 significantly lower in response patients than that in nonresponse patients (*p* = 0.040) based on real-time PCR. Patients with higher expression level of FZD8 with 75% stratification have significantly higher reduction in the questionnaire score (*p* = 0.010), and the area under the curve (AUC) was 0.765 (95%CI = 0.593–0.936; *p* = 0.014).

**Conclusions:**

FZD8 might perform the sensitive biomarker for predicting the effectiveness of XFZYC. However, further prospective cohort study was warranted to confirm the exact specificity and sensitivity of this biomarker.

## 1. Introduction

Within the framework of Traditional Chinese Medicine (TCM) theory, TCM practitioners always prescribe the herbal formula or acupuncture therapy according to the differentiation of TCM Zheng [1]. Hence, the diagnosis criteria of a sort of specific Zheng were often included in the inclusion criteria for a randomized clinical trial (RCT) in terms of Research and Development (R&D) of TCM new drug, in order to target the population with that kind of Zheng. However, in one of our previous clinical trials to investigate the effects of Xue-Fu-Zhu-Yu capsules (XFZYC), a kind of herbal medicine approved by the China Food and Drug Administration (CFDA), for patients with Qi Stagnation and Blood Stasis (QS&BS), a sort of typical Zheng, where QS&BS diagnosis criteria has been used for participants inclusion, the data of primary outcome in the XFZYC group failed to be within normality and the scatter diagram demonstrated that certain participants receiving XFZYC showed response in effectiveness while some participants indicated nonresponse. This abnormal distribution directly affected the statistical inference of the data and the interpretation of the results of the clinical trial, which might lead to the negative conclusion of the trial.

One of the reasons why there existed both response and nonresponse participants in the treatment group was that the QS&BS diagnosis criteria currently used in the trial was not adequate enough to target the exact population of only response patients. It has been reported that herbal medicine featured multicomponents and corresponding multitargets to perform the healing function [2], and a network pharmacology study of XFZYC for QS&BS predicted that there were 6 targets mostly possible for the merged network and target fishing [3]. Hence, we hypothesize that some patients with QS&BS are sensitive to the targets of XFZYC while others even with the same Zheng diagnosed by QS&BS criteria are insensitive to those targets, which might be the profound mechanism of the phenomenon of both response and nonresponse. Sensitive biomarker is just a sort of physiological substance in human body that when present in abnormal amounts in the serum may indicate the presence of disease or predict to what extent patients could respond to the therapy [4]. Therefore, to find the sensitive biomarker having the capacity to predict the effectiveness of XFZYC is crucial to narrow the scope of targeted population to the exact response population in the clinical trial.

The genetic profile of an individual provides information on what could theoretically happen in the body, while protein expression profiles are representative of the processes that are active in the body at the time-point of determination [5]. Of note, ribonucleic acid (RNA) builds the connection between deoxyribonucleic acid (DNA) and proteins, which features the interaction of genotype and environment. Among the different types of RNAs, messenger RNAs (mRNAs) are transcripts of DNA and the information they carry is transferred by their translation into proteins [6]. Long noncoding RNAs (lncRNAs) are a class of non-protein-coding RNAs of >200 nucleotides in length, and they are known to have roles in controlling chromatin structure, transcriptional regulation, and posttranscriptional processing [7, 8]. Furthermore, circular RNAs (circRNAs), which have drawn an increasing amount of attention recently, are a type of noncanonical form of alternative splicing and are more stable than linear RNAs [9, 10]. Therefore, the objective of this study was to identify the sensitive biomarker to predict the effectiveness of XFZYC by nested case-control design through transcriptome next-generation sequencing and real-time PCR in terms of RNAs.

## 2. Materials and Methods

### 2.1. Patients and Samples for RNA Sequencing

The protocol of the present study was approved by the Ethics Committee of Guang'anmen Hospital (Beijing, China). In our previous RCT, eligible participants fulfilled the diagnostic criteria for QS&BS pattern whose specificity and sensitivity of diagnosis are 81.91% and 80.35%, respectively [11]; the intervention group was treated with XFZYC, and the control group was placebo with 6 capsules of XFZYC or placebo 2 times daily and the duration was 7 weeks; the primary outcome was the change in the patient-reported QS&BS pattern questionnaire score from baseline to the end of the 7-week intervention. This questionnaire is a well-validated, multidimensional measurement of overall severity of QS&BS pattern [12]. The protocol of this previous trial was registered at the ClinicalTrials.gov (ID: NCT03091634).

This nested case-control study was designed and conducted based on the previous RCT. This study included 5 patients with response to XFZYC in the treatment group (response group), 5 patients with no response to XFZYC also in the treatment group (nonresponse group), and 5 patients in the control group treated with placebo (placebo group) who presented at Guang'anmen Hospital affiliated to the China Academy of Chinese Medical Sciences (Beijing, China) between June 2017 to August 2018 and participated in our previous RCT. Response was identified with reduction in this questionnaire score of >30%, while nonresponse with <30%. Blood sample was collected in 5 participants with response to XFZYC before the treatment (group Case1_before) and after the treatment (group Case1_after), 5 participants with no response to XFZYC before the treatment (group Case2_before), and 5 participants treated with placebo before the treatment (group Control_before) and after the treatment (group Control_after). From each of the patients, blood samples (3-4 ml) were collected in EDTA-containing tubes. Informed consent was provided by all of the participants prior to the study.

### 2.2. RNA Isolation and Library Preparation

RNA degradation and contamination were monitored on 1% agarose gels. RNA purity was conﬁrmed using the Agencourt AMPure XP (cat. no. A63881; Beckman Coulter, Brea, CA, USA). RNA integrity was evaluated using the Agilent 2100 bioanalyzer (Agilent Technologies, Inc., Santa Clara, CA, USA). The sample with RNA integrity number ≥7 was subjected to the subsequent analysis. The library was constructed using TruSeq-stranded total RNA with Ribo-Zero Gold (cat. no. 15021048; Illumina, Inc., San Diego, CA, USA) according to the manufacturer's instructions.

### 2.3. Sequencing of RNAs and DE RNA Analysis

The library was sequenced on the Illumina sequencing platform HiSeqTM 2500, and 150 bp/125 bp paired-end reads were generated. Trimmomatic software (version 0.36) [13] was used to further filter the quality of the raw data generated from the sequencing to get the high-quality clean reads, then Hierarchical Indexing for Spliced Alignment of Transcripts (version 0.1.6-beta) [14] was used to align clean reads to the reference of human beings, Stringtie software (version 1.3.5) [15] was used to assemble the reads so that the transcript was spliced. The spliced transcription products of each sample were combined and screened as lncRNAs with Cuffmerge program in the Cufflinks software package (version 2.2.1). All transcripts that overlapped with known mRNAs, other noncoding RNA, and non-lncRNA were discarded. Next, the transcripts longer than 200 bp and >2 exons were obtained, and CPC (version 0.9-r2) [16], PLEK (version 1.2) [17], CNCI (version 1.0) [18], and Pfam (version 30) [19] software packages were used to predict transcripts with coding potential. The characteristics (including length, type, and number of exons) of lncRNA were analyzed after screening by the Cuffcompare program [20] in the Cufflinks 2.2.1 software package. CircRNAs were identiﬁed using CIRI [21] and compared with the circBase [22] and with the database of CircAtlas 2.0 with species-focused integrated resource of *Homo sapiens* [23]. The estimate size factors function in R package DESeq [24] was used to normalize the counts, and nbinom test in the package was used to calculate the *p* value as well as fold change for the difference comparison. The difference comparison was conducted between group Case1_before and Case1_after, between Control_before and Control_after, and between Case1_before and Case2_before, to get 3 sets of differential-expressed (DE) RNAs for further analysis of sensitive biomarker. DE RNAs were identified if *p* value ≤ 0.05 and fold change ≥2.

### 2.4. GO Annotations and KEGG Pathway Analysis

Gene ontology (GO) annotations and Kyoto Encyclopedia of Genes and Genomes (KEGG) pathway analysis were performed to investigate the possible roles of the DE RNAs. GO annotations were performed to identify regulatory networks of DE genes (http://geneontology.org). KEGG analysis was also performed to explore the enriched pathways of the DE RNAs based on the KEGG database (http://www.genome.jp/kegg/). To reveal the role and interactions among the DE RNAs, an RNA regulatory network was constructed using Cytoscape software version 3.2.1 (http://www.cytoscape.org/) based on the prediction and interaction analysis by miRWalk 2.0 (http://zmf.umm.uni-heidelberg.de/apps/zmf/mirwalk2/) and starBase 3.0 (http://starbase.sysu.edu.cn/).

### 2.5. Real-Time PCR and Sensitivity Analysis

A series of clinical data and blood sample were collected from 30 participants with both response and nonresponse to XFZYC in treatment group before intervention. The expression levels of the core RNAs in the regulatory network were confirmed by quantitative real-time PCR. Sensitive biomarker analysis was conducted to identify the RNA that could predict the effectiveness of XFZYC among patients diagnosed with QS&BS and to further validate the association between DE RNAs and response degree to XFZYC.

### 2.6. Statistical Analysis

The data regarding the clinical characteristics of the participants are expressed as the mean ± standard error of the mean, and the corresponding results were statistically analyzed using one-way analysis of variance. The results of DE RNAs in the comparison of Case1_before vs Case1_after, Control_before vs Control_after, and Case1_before vs Case2_before were analyzed by the algorithm of DESeq2 in R software [24], and overlap DE RNAs between these three comparisons were identified by Venn's diagram [25]. 2^−Δ*Ct*^ data generated from real-time PCR were used to compare the confirmed DE RNAs [26]. The multiple regression analysis was used to determine the independent predictive factors for response of XFZYC. Receiver operating characteristics (ROC) curve was used to analyze the predictive value of the biomarker. All statistical analyses were performed using R software, and *p* < 0.05 was considered to indicate a statistically significant difference.

## 3. Results

### 3.1. Clinical Characteristics of Participants

The distributions of clinical characteristics between the selected participants from treatment group and placebo group are listed in Table 1. The 2 groups were matched in terms of sex, age, race, Body-mass index, and severity of the pattern, and there were no significant differences in terms of self-reported coexisting illness and routine medication between the groups (*p* > 0.05).

### 3.2. DE RNAs and Their Overlap among Groups

**Figure 1:**
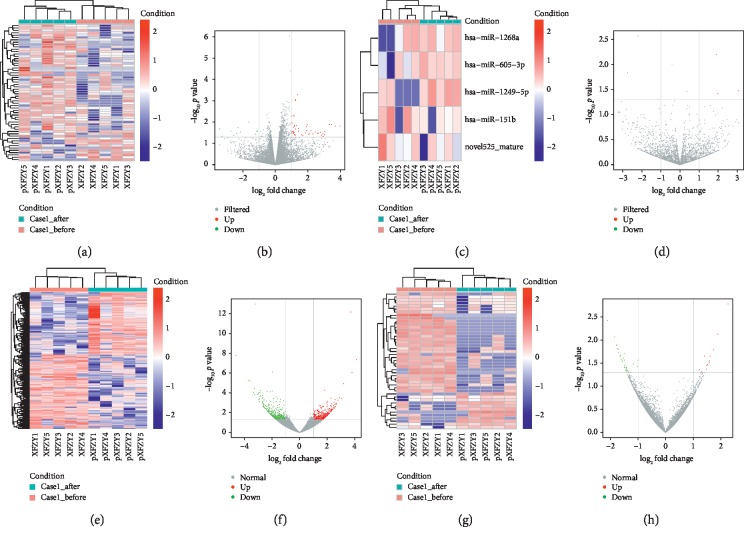
Clustering map and volcano plot of DE RNAs for comparison of Case1_before and Case1_after. (a, c, e, g) Heat maps present the hierarchical clustering of DE RNAs with upregulated RNAs marked in red and downregulated ones marked in blue. (b, d, f, h) Volcano plots indicate up- and downregulated RNAs with |log_2_FC| > 1 and *p* < 0.05, where upregulated RNA was marked in red and downregulated RNA was marked in green. From left to right, mRNA, miRNA, lncRNA, and circRNA are shown in sequence.

**Figure 2:**
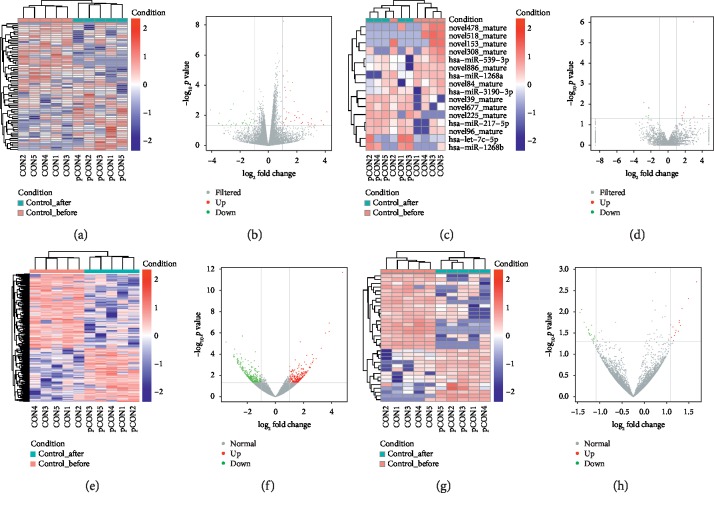
Clustering map and volcano plot of DE RNAs for comparison of Control_before and Control_after. (a, c, e, g) Heat maps present the hierarchical clustering of DE RNAs with upregulated RNA marked in red and downregulated RNA marked in blue. (b, d, f, h) volcano plots indicate up- and downregulated RNAs with |log_2_FC| > 1 and *p* value < 0.05, where upregulated RNA was marked in red and downregulated was marked in green. From left to right, mRNA, miRNA, lncRNA, and circRNA are shown in sequence.

**Figure 3:**
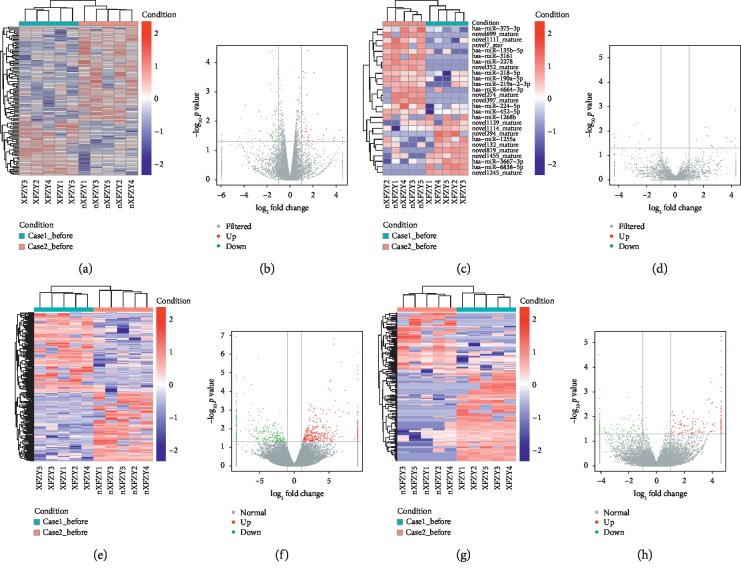
Clustering map and volcano plot of DE RNAs for comparison of Case1_before and Case2_before. (a, c, e, g) Heat maps present the hierarchical clustering of DE RNAs with upregulated RNA marked in red and downregulated RNA marked in blue. (b, d, f, h) Volcano plots indicate up- and downregulated RNAs with |log_2_FC| > 1 and *p* < 0.05, where upregulated RNA was marked in red and downregulated RNA was marked in green. From left to right, mRNA, miRNA, lncRNA, and circRNA are shown in sequence.

The results of the DE RNAs in the comparisons were as follows: a total of 58 DE mRNAs (46 upregulated and 12 downregulated), 17 DE miRNAs (8 upregulated and 9 downregulated), 691 DE lncRNAs (317 upregulated and 374 downregulated), and 161 DE circRNAs (95 upregulated and 66 downregulated) were identified with fold change >2 and *p* value <0.05 in comparison between Case1_before and Case1_after, respectively. In comparison between Control_before and Control_after, a total of 71 DE mRNAs (37 upregulated and 34 downregulated), 16 DE miRNAs (9 upregulated and 7 downregulated), 616 DE lncRNAs (259 upregulated and 357 downregulated), and 34 DE circRNAs (14 upregulated and 20 downregulated) were identified with fold change >2 and *p* value <0.05, respectively. After removing the overlapped DE RNA between Case1_before vs Case1_after and Control_before vs Control_after, the unique DE RNA in Case1_before vs Case1_after included 1156 DE RNAs, 388 miRNAs, 1954 lncRNAs, and 560 circRNAs, which were associated the mechanism of XFZYC and composed the targeted potential biomarkers for further real-time PCR. In comparison between Case1_before and Case2_before, a total of 107 DE mRNAs (39 upregulated and 68 downregulated), 45 DE miRNAs (12 upregulated and 33 downregulated), 721 DE lncRNAs (383 upregulated and 338 downregulated), and 242 DE circRNAs (148 upregulated and 94 downregulated) were identified with fold change >2 and *p* value < 0.05, which also formed the potential biomarkers for further real-time PCR. The top 10 upregulated and top 10 downregulated DE mRNAs, miRNAs, lncRNAs, and circRNAs are listed in Supplementary Tables [Supplementary-material supplementary-material-1], [Supplementary-material supplementary-material-1], [Supplementary-material supplementary-material-1], and [Supplementary-material supplementary-material-1], respectively, and the clustering maps as well as volcano plot was displayed in Figures 1–3 in terms of Case1_before vs Case1_after, Control_before vs Control_after, and Case1_before vs Case2_before, respectively.

### 3.3. Functional Prediction and Merged Interactions of RNAs

**Figure 4:**
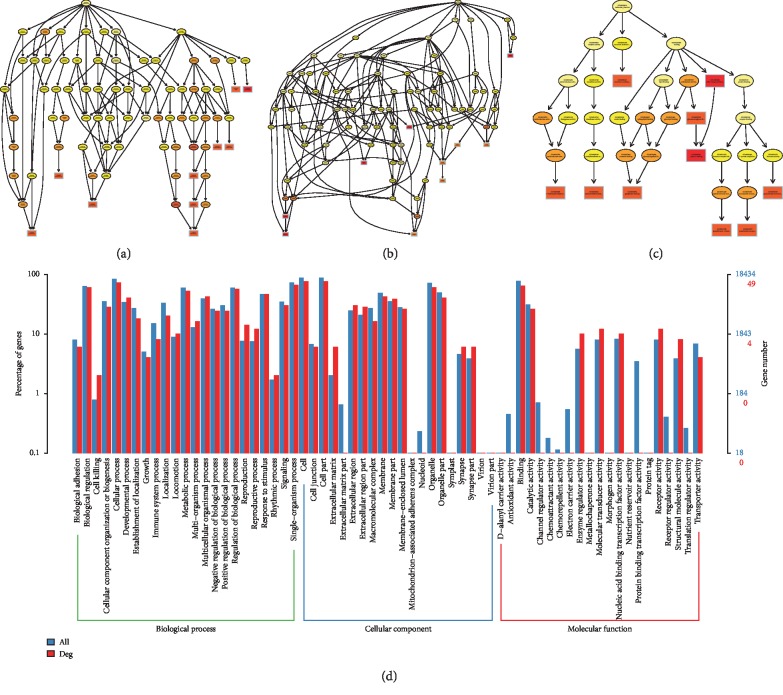
GO analysis of DE mRNAs. The GO enrichment analysis of DE RNAs is represented by directed acyclic graph in (a), (b), and (c), and the GO terms and number are shown by histogram in (d).

**Figure 5:**
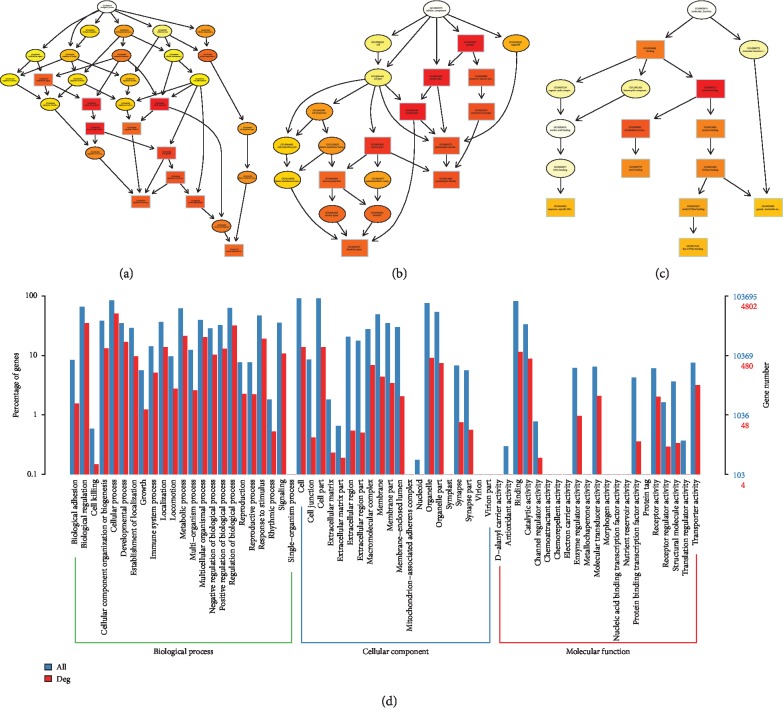
GO analysis of DE miRNAs. The GO enrichment analysis of DE RNAs is represented by directed acyclic graph in (a), (b), and (c), and the GO terms and number are shown by histogram in (d).

**Figure 6:**
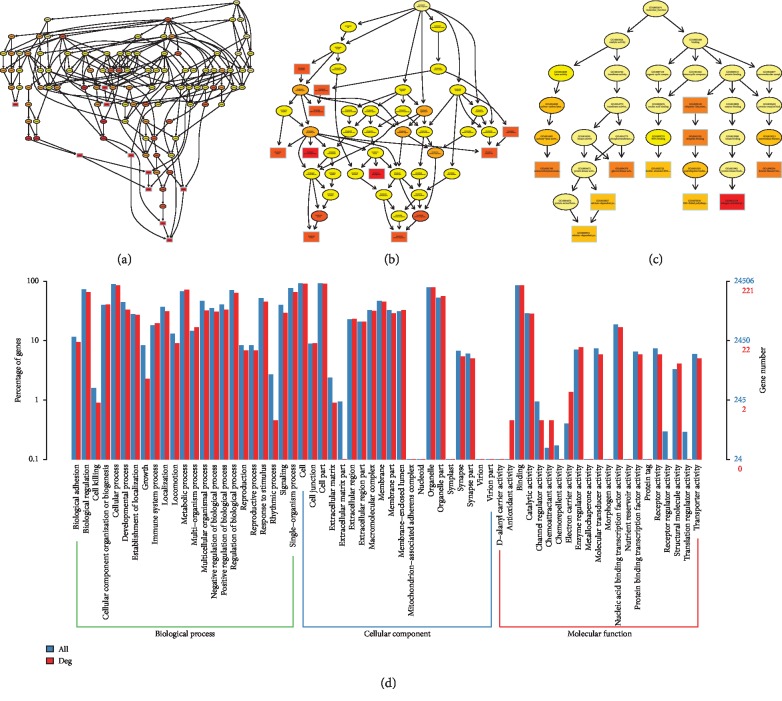
GO analysis of DE lncRNAs. The GO enrichment analysis of DE RNAs is represented by directed acyclic graph in (a), (b), and (c), and the GO terms and number are shown by histogram in (d).

**Figure 7:**
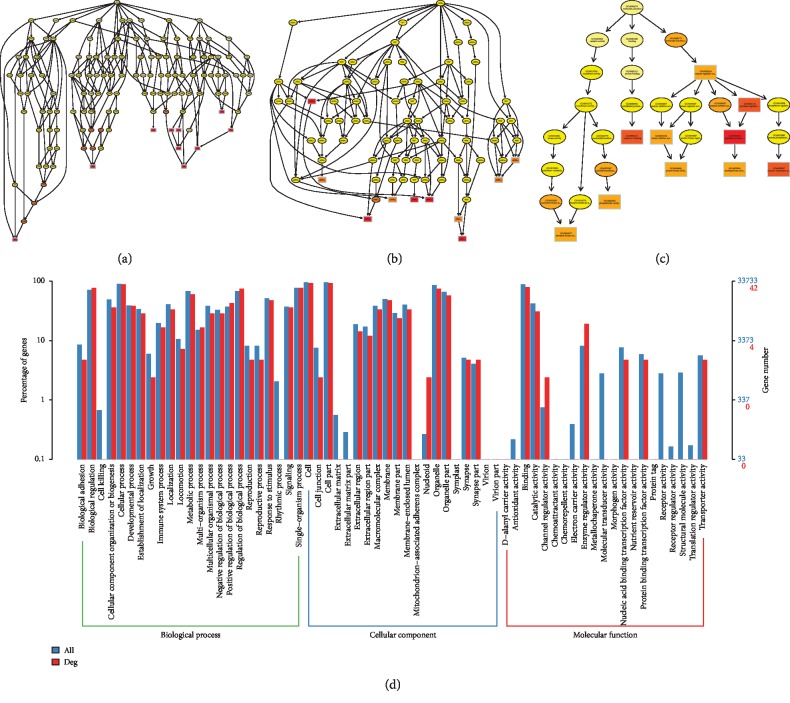
GO analysis of DE circRNAs. The GO enrichment analysis of DE RNAs is represented by directed acyclic graph in (a), (b), and (c), and the GO terms and number are shown by histogram in (d).

**Figure 8:**
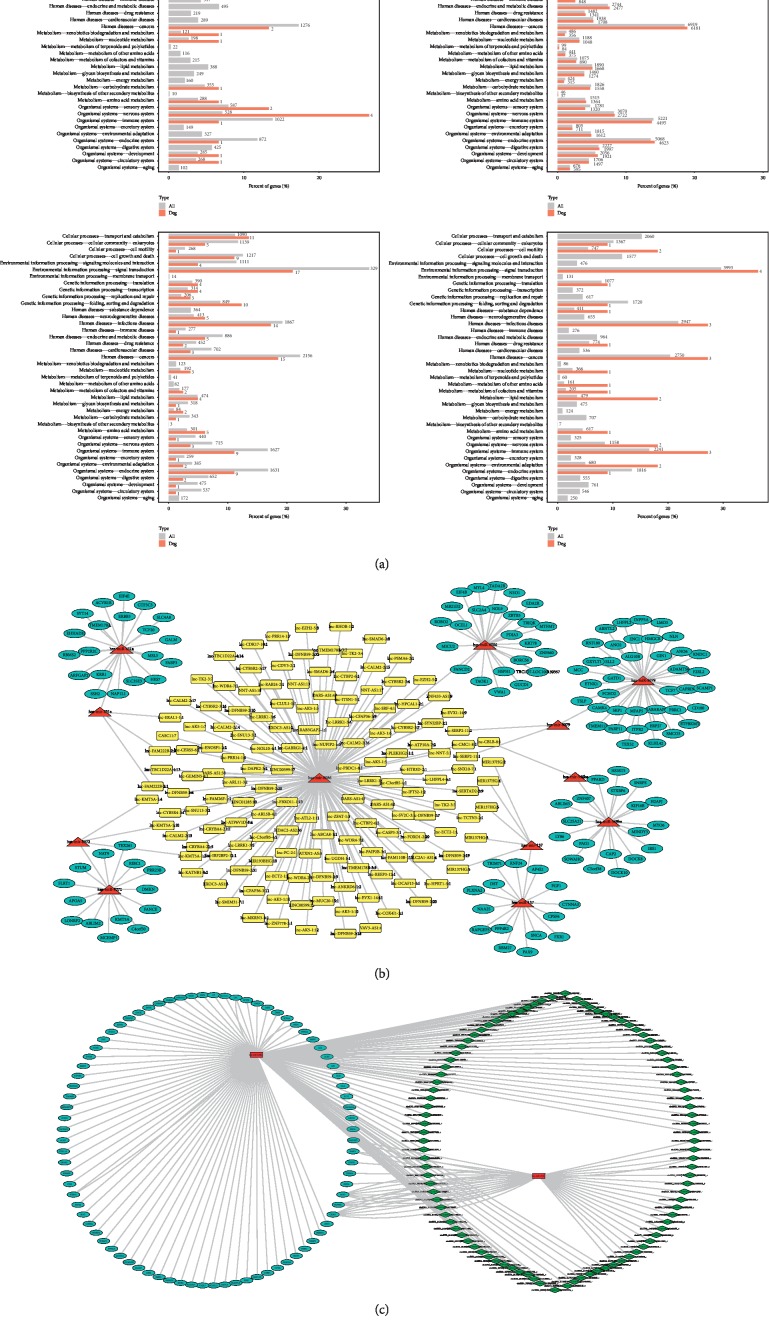
(a) Enrichment analysis in KEGG pathways of DE mRNAs, miRNAs, lncRNAs, and circRNAs, respectively. (b) The lncRNA-miRNA-mRNA interaction network of DE RNAs. (c) The circRNA-miRNA-mRNA interaction network of DE RNAs. The enriched pathways and related gene number are shown in the histogram. The miRNAs are marked in red, lncRNAs are marked in yellow, circRNAs are marked in green, and mRNAs are marked in blue.

To further determine the possible functions of the DE RNAs and to investigate the association between these functions and underlying mechanisms in the biological networks, significant GO terms in the categories of biological process, cellular component, and molecular function are shown Figures 4–7 in terms of mRNA, miRNA, lncRNA, and circRNA, respectively, and enrichment analysis based on KEGG database is shown in Figure 8(a) in terms of mRNA, miRNA, lncRNA, and circRNA, respectively. The DE RNAs were enriched in KEGG pathways such as glyoxylate and dicarboxylate metabolism, glycine, serine and threonine metabolism, neomycin, kanamycin and gentamicin biosynthesis, lipoic acid metabolism, steroid biosynthesis, and one carbon pool by folate. The interaction analysis of DE RNAs is shown in Figure 8(b) in terms of the merged network of lncRNA-miRNA-mRNA and in Figure 8(c) in terms of the network of circRNA-miRNA-mRNA.

### 3.4. Real-Time PCR and Sensitive Biomarker Prediction

To validate the association between DE RNAs and response to XFZYC, a set of 30 patients with both response and nonresponse was further analyzed by using quantitative real-time PCR. Consistently, we found the expression level of FZD8 was significantly higher in response patients than that in nonresponse patients (*p*=0.041) and circRNA_13799 significantly lower in response patients than that in nonresponse patients (*p*=0.040) as is shown in Table 2, confirming that the higher expression of FZD8 and lower expression of circRNA_13799 are associated with a better response to XFZYC. Furthermore, analyzing the reduction in questionnaire score of these 30 patients after 50%, 75%, and 90% stratification by the level of FZD8 and circRNA_13799 revealed that only patients with higher expression level of FZD8 with 75% stratification have significantly higher reduction in questionnaire score (*p*=0.010). Nevertheless, multiple regression analysis showed that only the level of FZD8 could be an independent predictive factor for response to XFZYC (*p*=0.0003). To evaluate this predictive value, the ROC curve was used to analyze the sensitivity and specificity. The area under the curve (AUC) was 0.765 (95%CI = 0.593–0.936; *p*=0.014). These findings suggested that increased expression of FZD8 may be responsible for high sensitivity to XFZYC and predicting the response in QS&BS patients.

## 4. Discussion

In the present study, expression profiles of mRNA, miRNA, lncRNA, and circRNA were obtained by next-generation sequencing and compared in before-treatment and after-treatment of participants selected in the randomized clinical trials and also compared in response and nonresponse participants within the nested case-control design, and then sensitive biomarker for predicting the effectiveness of XFZYC was analyzed based on the functional prediction and merged interactions of the identified DE RNAs. Patients with higher expression level of FZD8 with 75% stratification have significantly higher reduction in questionnaire score, indicating a better response.

XFZYC is a common Chinese patent medicine approved by CFDA for treating QS &BS pattern. However, there were both response and nonresponse participants in the treatment group in our previous randomized clinical trial, which indicated that the diagnosis criteria for QS&BS pattern in the clinical trial should be further narrowed, that is, the indications of the population for XFZYC should be clarified and specified so that the population with response could be targeted in the clinical trial and in the clinical practice. Adding the objective sensitive biomarker of FZD8 with 75% stratification to the diagnosis criteria composed of only symptoms and signs we currently used might specifically narrow the scope of targeted population and then improve the effectiveness of XFZYC.

The function annotation of the DE RNAs indicated that the sensitive biomarker FZD8 is a member of the frizzled gene family and a sort of receptor for Wnt proteins, and the component of the Wnt-Fzd-LRP5-LRP6 complex could trigger beta-catenin signaling through inducing aggregation of receptor-ligand complexes into ribosome-sized signalosomes [27]. This signaling pathway could lead to the activation of disheveled proteins, inhibition of GSK-3 kinase, nuclear accumulation of beta-catenin, and activation of Wnt target genes. Moreover, FZD8 also gets involved in PKC and calcium fluxes pathway. Both these two pathways seem to involve interactions with G-proteins and to participate in the process of transduction and intercellular transmission during tissue morphogenesis and pathology of inflammation [28]. Bioinformatics analysis of the RNA sequencing results of the present study indicated that DE RNAs were most significantly enriched in pathways including Wnt signaling pathway, glyoxylate and dicarboxylate metabolism, glycine, serine and threonine metabolism, and homologous recombination. These results imply that the pharmacological functions of XFZYC is associated with both pathogenesis of QS&BS associated diseases such as coronary heart disease, digestive diseases, osteoporosis, and type 2 diabetes [29] and vital metabolism process [30]. In the lncRNA-miRNA-mRNA and circRNA-miRNA-mRNA networks, the core regulator hsa-miR-137 is closely associated with the nerve system [31], and hsa-miR-451a performs the function in the process of myocardial lesions [32].

To the best of our knowledge, the present study was the first to explore the sensitive biomarker for effectiveness of XFZYC and it was attempted to interpret the phenomenon of existing of both response and nonresponse patients, from the perspective of RNAs, which was determined by next-generation sequencing and real-time PCR within the design of nested case control. It is worth mentioning that this sensitive biomarker could be used in future clinical trial in terms of inclusion criteria and that the design of nested case-control in this study might be implemented to discover biomarker of a sort of Chinese herbal formulae in further studies. However, one of the limitations of the present study is the lack of validation of the sensitive biomarker in prospective cohort study to confirm the specificity and sensitivity of this biomarker. Furthermore, the sensitive biomarker associated with the pharmacological mechanism of XFZYC has not been tested in animal or cell study because of lack of animal or cell model of QS&BS pattern.

## 5. Conclusion

FZD8 may act as sensitive biomarker for predicting the effectiveness of XFZYC. However, further prospective cohort study was warranted to confirm the exact specificity and sensitivity of this biomarker.

## Figures and Tables

**Table 1:** Baseline characteristics of the study participants^*∗*^.

Variable	Response group (*n* = 5)	Nonresponse group (*n* = 5)	Placebo group (*n* = 5)
Sex, *n*			
Male	3	4	3
Age, yr			
Mean age (SD)	49.80 ± 6.68	43.80 ± 18.32	42.60 ± 13.22
Race, *n*			
Han	5	5	5
Body-mass index^‡^	21.39 ± 3.79	22.90 ± 0.56	24.34 ± 4.10
Severity of QS&BS pattern^§^	34.40 ± 10.11	31.60 ± 6.03	34.00 ± 5.34
Self-reported coexisting illness, *n* (%)			
Atherosclerosis	1	0	2
Digestive system disease	3	1	0
Reproductive system disease	3	3	1
Osteoarthritis	1	2	1
Hypertension	0	1	1
Diabetes	0	0	0
Routine medications, *n* (%)			
Antiplatelet drugs	0	0	1
Antihypertensive drug	0	1	1
Statins	0	0	0
Oral hypoglycemic drugs	0	0	0

XFZYC = Xue-Fu-Zhu-Yu Capsules; QS&BS = Qi Stagnation and Blood Stasis. ^*∗*^Plus-minus values are mean ± SD unless otherwise noted. ^‡^Body mass index is the weight in kilograms divided by the square of the height in meters. ^§^Severity of QS&BS pattern is assessed by the scores of the items in diagnostic criteria for QS&BS pattern. Scores range from 0 to 51, with higher scores indicating more severe pattern of diagnosis.

**Table 2:** Real-time PCR results.

RNA name	2^−Δ*Ct*^ (MD ± SD)		*P* value
	Response	Nonresponse	
OR2L5	0.000217 ± 0.000279	0.000117 ± 0.000051	0.163
CALN1	0.000110 ± 0.000153	0.000075 ± 0.000062	0.445
IL3RA	0.000621 ± 0.000195	0.000678 ± 0.000204	0.448
GALNT13	0.000138 ± 0.000196	0.000051 ± 0.000027	0.124
FZD8	0.000191 ± 0.000147	0.000109 ± 0.000040	0.041
SLC25A6	0.001081 ± 0.000770	0.000845 ± 0.000337	0.312
PRR25	0.000054 ± 0.000042	0.000039 ± 0.000025	0.242
circRNA_13799	0.000073 ± 0.000075	0.000031 ± 0.000020	0.040
hsa-miR-4664-3p	0.000444 ± 0.000203	0.000499 ± 0.000168	0.438
hsa-miR-190a-5p	0.000003 ± 0.000001	0.000003 ± 0.000001	0.979
hsa-miR-3667-3p	0.000048 ± 0.000032	0.000049 ± 0.000018	0.945
hsa-miR-1268b	0.007495 ± 0.002283	0.006612 ± 0.001168	0.214

MD, mean difference; SD, standard deviation.

## Data Availability

The data used to support the findings of this study are available from the corresponding author upon request.

## Supplementary Materials

Supplementary Materials The top 10 upregulated and top 10 downregulated DE mRNAs, miRNAs, lncRNAs, and circRNAs with the comparison of Case1_before vs Case1_after, Control_before vs Control_after, and Case1_before vs Case2_before.
