# Supplementary material for: Sensitive Biomarker Analysis of Xue-Fu-Zhu-Yu Capsule for Patients with Qi Stagnation and Blood Stasis Pattern: A Nested Case-Control Study
Source: Evid Based Complement Alternat Med. 2019 Nov 18;2019:7182865. doi: 10.1155/2019/7182865 (PMC6885824; doi:10.1155/2019/7182865)
Supplement: Supplementary Materials — The top 10 upregulated and top 10 downregulated DE mRNAs, miRNAs, lncRNAs, and circRNAs with the comparison of Case1_before vs Case1_after, Control_before vs Control_after, and Case1_before vs Case2_before. [file 7182865.f1.docx]

**Supplymentary Table 1. Detailed information of the top 10 upregulated and 10 downregulated mRNAs.**

| Case1_before vs Case1_after | | | | |
| --- | --- | --- | --- | --- |
| Gene name | Fold change | log_2_(Fold change) | p value | Regulation |
| ADAM29 | 0.093219672 | -3.42322176 | 0.020622553 | Down |
| LOC390877 | 0.093402367 | -3.420397072 | 0.02098899 | Down |
| THAP10 | 0.102630064 | -3.28447468 | 0.046396546 | Down |
| KLHL30 | 0.149629996 | -2.740528679 | 0.026328171 | Down |
| SHROOM3 | 0.168729137 | -2.567218971 | 0.042716556 | Down |
| REG4 | 0.190973028 | -2.388559198 | 0.034723831 | Down |
| ASMTL | 0.201453744 | -2.311479479 | 0.018092325 | Down |
| ANOS1 | 0.223337135 | -2.162704942 | 0.029552394 | Down |
| DEFA1 | 0.322440293 | -1.632896055 | 0.010169181 | Down |
| C19orf33 | 0.428049006 | -1.22415212 | 0.036901998 | Down |
| SHANK1 | 6.83278956 | 2.772474695 | 0.029998863 | Up |
| PCOLCE2 | 7.213494152 | 2.850698257 | 0.04145831 | Up |
| ECT2L | 7.632768073 | 2.932206355 | 0.035593368 | Up |
| ACTRT3 | 7.67751937 | 2.940640247 | 0.046118747 | Up |
| FAM170B | 8.199619248 | 3.035556919 | 0.038976505 | Up |
| BRSK2 | 8.330946702 | 3.058480448 | 0.026663449 | Up |
| SOX30 | 10.03390774 | 3.326811672 | 0.012878309 | Up |
| DRD3 | 11.06820857 | 3.46834983 | 0.012603236 | Up |
| KLK14 | 13.03502286 | 3.704321208 | 0.015269048 | Up |
| LOC101928095 | 15.78878718 | 3.98082845 | 0.015142324 | Up |
| Control_before vs Control_after | | | | |
| Gene name | Fold change | log2(Fold change) | p value | Regulation |
| TBC1D3H | 0.060251786 | -4.052852193 | 0.004292176 | Down |
| CLMP | 0.087097732 | -3.521221044 | 0.032691492 | Down |
| PPP1R36 | 0.089254939 | -3.485924182 | 0.036005209 | Down |
| ZP1 | 0.091945687 | -3.443074281 | 0.0071718 | Down |
| CDRT1 | 0.106869202 | -3.226081949 | 0.045470686 | Down |
| 10-Mar | 0.128368164 | -2.96164064 | 0.03951291 | Down |
| PTPRD | 0.144338574 | -2.792471189 | 0.025241055 | Down |
| RND1 | 0.152674058 | -2.71147315 | 0.021155396 | Down |
| IL3RA | 0.160756271 | -2.637053082 | 0.004043978 | Down |
| NFATC4 | 0.164441184 | -2.604356432 | 0.039884985 | Down |
| GRIN2D | 5.316544968 | 2.410488995 | 0.028915635 | Up |
| STON1-GTF2A1L | 5.447443721 | 2.445579387 | 0.008768349 | Up |
| LNX1 | 7.001838198 | 2.807733724 | 0.03053833 | Up |
| FAM198A | 7.188176822 | 2.845625898 | 0.041770429 | Up |
| DACT3 | 8.187304469 | 3.033388547 | 0.036871372 | Up |
| XG | 8.355869629 | 3.062789983 | 0.016821828 | Up |
| NOSTRIN | 9.817789273 | 3.295398201 | 0.043954172 | Up |
| WDR72 | 13.36138213 | 3.739997345 | 0.022484402 | Up |
| SUN3 | 14.94058669 | 3.901164896 | 0.041526246 | Up |
| MEIS3 | 17.48670742 | 4.128186764 | 0.005807305 | Up |
| Case1_before vs Case2_before | | | | |
| Gene name | Fold change | log2(Fold change) | p value | Regulation |
| OR2L5 | 0.043546824 | -4.521288696 | 0.044261986 | Down |
| CALN1 | 0.047261962 | -4.403176664 | 0.03480864 | Down |
| IL3RA | 0.052116073 | -4.26212781 | 0.008057207 | Down |
| LOC102723728 | 0.067334993 | -3.892499745 | 0.002785888 | Down |
| GALNT13 | 0.069688951 | -3.84292626 | 0.025661495 | Down |
| TM4SF1 | 0.087044053 | -3.522110455 | 0.020370097 | Down |
| CCL8 | 0.106384483 | -3.232640359 | 0.044017525 | Down |
| SULT1A4 | 0.126019715 | -2.988278638 | 0.038754563 | Down |
| C19orf73 | 0.155133639 | -2.688416545 | 0.004305902 | Down |
| SHANK1 | 0.155742103 | -2.682769079 | 0.021928335 | Down |
| LOC100996720 | 4.205377853 | 2.072235433 | 0.000221644 | Up |
| FZD8 | 5.340871211 | 2.417075096 | 0.005660787 | Up |
| PEG3 | 5.652707931 | 2.498942156 | 0.042351373 | Up |
| CLDN10 | 7.268630021 | 2.861683473 | 0.041597958 | Up |
| TMC5 | 7.608932567 | 2.927694077 | 0.030708899 | Up |
| SNX7 | 8.980597843 | 3.166811489 | 0.018636844 | Up |
| SLC25A6 | 13.67879007 | 3.77386872 | 0.04491566 | Up |
| LOC101927345 | 14.55899407 | 3.863838774 | 0.03656306 | Up |
| PRR25 | 19.28117542 | 4.269121099 | 0.02087694 | Up |
| LOC390877 | 23.65832463 | 4.564276008 | 0.035918446 | Up |

**Supplymentary Table 2. Detailed information of the top 10 upregulated and 10 downregulated miRNAs.**

| Case1_before vs Case1_after | | | | | | | |  |
| --- | --- | --- | --- | --- | --- | --- | --- | --- |
| miRNA_id | Fold change | | log_2_(Fold change) | | p value | | Regulation |  |
| hsa-miR-1268b | 0.000392 | | -11.3161 | | 1.59E-18 | | Down |  |
| hsa-miR-6810-3p | 0.002157 | | -8.85662 | | 0.000375 | | Down |  |
| hsa-miR-6749-3p | 0.002589 | | -8.59359 | | 0.001279 | | Down |  |
| hsa-let-7g-3p | 0.003236 | | -8.27166 | | 0.004357 | | Down |  |
| hsa-miR-186-3p | 0.003236 | | -8.27166 | | 0.004357 | | Down |  |
| hsa-miR-3180-3p | 0.003236 | | -8.27166 | | 0.004357 | | Down |  |
| hsa-miR-337-5p | 0.003236 | | -8.27166 | | 0.004357 | | Down |  |
| hsa-miR-6813-3p | 0.003236 | | -8.27166 | | 0.004357 | | Down |  |
| hsa-miR-188-5p | 0.004314 | | -7.85662 | | 0.014845 | | Down |  |
| hsa-miR-2355-5p | 0.004314 | | -7.85662 | | 0.014845 | | Down |  |
| hsa-miR-218-5p | 17.39623 | | 4.120703 | | 0.001504 | | Up |  |
| hsa-miR-744-3p | 516.9387 | | 9.013849 | | 0.043766 | | Up |  |
| hsa-miR-627-5p | 568.6325 | | 9.151353 | | 0.03092 | | Up |  |
| hsa-miR-1298-5p | 620.3264 | | 9.276884 | | 0.021844 | | Up |  |
| hsa-miR-29b-1-5p | 672.0203 | | 9.392361 | | 0.015432 | | Up |  |
| hsa-miR-3934-5p | 672.0203 | | 9.392361 | | 0.015432 | | Up |  |
| hsa-miR-6837-3p | 878.7957 | | 9.779384 | | 0.003844 | | Up |  |
| hsa-miR-183-3p | 930.4896 | | 9.861846 | | 0.002716 | | Up |  |
| hsa-miR-2355-3p | 1654.204 | | 10.69192 | | 2.10E-05 | | Up |  |
| hsa-miR-1268a | 1809.285 | | 10.8212 | | 7.39E-06 | | Up |  |
| Control_before vs Control_after | | | | | | | |  |
| miRNA_id | | Fold change | | log_2_(Fold change) | p value | Regulation | |  |
| hsa-let-7c-5p | | 0.139934 | | -2.83718 | 0.008093 | Down | |  |
| hsa-miR-1268b | | 0.002751 | | -8.50588 | 9.33E-07 | Down | |  |
| hsa-miR-217-5p | | 0.16696 | | -2.58243 | 0.038101 | Down | |  |
| novel225_mature>novel435_mature | | 0.20205 | | -2.30721 | 0.043018 | Down | |  |
| novel39_mature | | 0.215969 | | -2.21111 | 0.015377 | Down | |  |
| novel677_mature | | 0.208418 | | -2.26245 | 0.014864 | Down | |  |
| novel96_mature | | 0.210146 | | -2.25053 | 0.035478 | Down | |  |
| hsa-miR-1268a | | 7.837102 | | 2.97032 | 9.63E-07 | Up | |  |
| hsa-miR-3190-3p | | 3.170521 | | 1.66472 | 0.047117 | Up | |  |
| hsa-miR-539-3p | | 3.092987 | | 1.629001 | 0.036014 | Up | |  |
| novel153_mature | | Inf | | Inf | 0.040091 | Up | |  |
| novel308_mature>novel1450_mature | | 16.19763 | | 4.017711 | 0.036781 | Up | |  |
| novel478_mature | | 8.158347 | | 3.028277 | 0.042474 | Up | |  |
| novel518_mature>novel1238_mature | | Inf | | Inf | 0.010258 | Up | |  |
| novel84_mature | | 3.548589 | | 1.827245 | 0.026328 | Up | |  |
| novel886_mature | | 4.094764 | | 2.03378 | 0.013742 | Up | |  |
| Case1_before vs Case2_before | | | | | | | | |
| miRNA_id | | Fold change | | log_2_(Fold change) | p value | | Regulation | |
| hsa-miR-4664-3p | | 0.0746947 | | -3.74285 | 0.0479523 | | Down | |
| hsa-miR-190a-5p | | 0.1052308 | | -3.248372 | 0.0169231 | | Down | |
| hsa-miR-219a-2-3p | | 0.140633 | | -2.829993 | 0.0058945 | | Down | |
| hsa-miR-135b-5p | | 0.1626522 | | -2.620138 | 0.004763 | | Down | |
| hsa-miR-452-5p | | 0.1700488 | | -2.55598 | 0.0281707 | | Down | |
| hsa-miR-218-5p | | 0.1927702 | | -2.375046 | 0.0158388 | | Down | |
| hsa-miR-375-3p | | 0.2783379 | | -1.845091 | 0.0219434 | | Down | |
| novel1111_mature | | 0.2883917 | | -1.793898 | 0.0213331 | | Down | |
| hsa-miR-224-5p | | 0.4261362 | | -1.230614 | 0.0374351 | | Down | |
| hsa-miR-1255a | | 2.8690316 | | 1.5205639 | 0.0231208 | | Up | |
| hsa-miR-3667-3p | | 4.4625972 | | 2.1578836 | 0.0071892 | | Up | |
| novel132_mature>novel388_mature | | 4.7639947 | | 2.2521718 | 0.0158907 | | Up | |
| novel1114_mature | | 4.8655985 | | 2.2826173 | 0.0158057 | | Up | |
| novel1129_mature>novel1133_mature | | 5.8723286 | | 2.5539327 | 0.0391314 | | Up | |
| hsa-miR-1268b | | 6.1781214 | | 2.6271682 | 0.0436308 | | Up | |
| novel1455_mature | | 12.47143 | | 3.640555 | 0.018905 | | Up | |
| novel819_mature | | 15.740968 | | 3.9764523 | 0.0270937 | | Up | |

**Supplymentary Table 3. Detailed information of the top 10 upregulated and 10 downregulated lncRNAs.**

| Case1_before vs Case1_after | | | | | |  |
| --- | --- | --- | --- | --- | --- | --- |
| lncRNA_id | type | Fold change | log_2_(Fold change) | p value | Regulation |  |
| NUTM2A-AS1:24 | intergenic | 17.42138276 | 4.122787232 | 4.43E-08 | Up |  |
| TSPOAP1-AS1:28 | intergenic | 13.9431653 | 3.801486206 | 8.84E-07 | Up |  |
| TCONS_00000342 | genic | 13.18091759 | 3.720378902 | 6.80E-13 | Up |  |
| NEAT1:18 | intergenic | 9.195331529 | 3.200901591 | 1.22E-05 | Up |  |
| lnc-ROPN1L-4:1 | unknown | 8.339223543 | 3.059913062 | 0.000363917 | Up |  |
| lnc-CHRNA7-5:2 | unknown | 7.48238376 | 2.903497961 | 0.000301944 | Up |  |
| lnc-TXNDC5-5:1 | unknown | 6.968822606 | 2.800914931 | 0.000563406 | Up |  |
| lnc-TMEM178A-6:4 | unknown | 6.407160964 | 2.679685235 | 0.001368023 | Up |  |
| OSER1-AS1:6 | unknown | 6.403537059 | 2.678869013 | 0.001248443 | Up |  |
| lnc-AGPS-6:6 | unknown | 6.089044591 | 2.606215878 | 0.002582169 | Up |  |
| lnc-CEP19-1:1 | intergenic | 0.128065989 | -2.965040705 | 0.000102056 | Down |  |
| lnc-RPIA-4:1 | unknown | 0.126026156 | -2.988204901 | 0.00013171 | Down |  |
| lnc-CDK12-1:1 | unknown | 0.120565439 | -3.052111694 | 0.000144704 | Down |  |
| TCONS_00043639 | unknown | 0.107527612 | -3.217220916 | 1.05E-13 | Down |  |
| lnc-HIST1H3D-2:1 | unknown | 0.1047092 | -3.255539884 | 0.00014162 | Down |  |
| lnc-CAB39L-4:3 | unknown | 0.103241067 | -3.275911142 | 3.81E-05 | Down |  |
| ZEB1-AS1:14 | intergenic | 0.099748909 | -3.325555133 | 9.50E-05 | Down |  |
| lnc-RTP5-8:1 | unknown | 0.083143326 | -3.588255734 | 6.46E-06 | Down |  |
| lnc-HIST1H2BI-2:2 | unknown | 0.077217444 | -3.694929397 | 5.92E-06 | Down |  |
| lnc-CWC15-1:2 | unknown | 0.0416117 | -4.586866971 | 1.58E-08 | Down |  |
| Control_before vs Control_after | | | | | | |
| lncRNA_id | type | Fold change | log_2_(Fold change) | p value | Regulation | |
| lnc-TTC30B-7:7 | unknown | 0.088633914 | -3.49599737 | 7.09E-06 | 0.01661478 | |
| lnc-RPIA-2:6 | unknown | 0.126691956 | -2.980603168 | 0.000147691 | 0.151512458 | |
| lnc-HOMER3-3:1 | unknown | 0.129421837 | -2.949847037 | 0.00018303 | 0.166903332 | |
| lnc-CHL1-7:45 | unknown | 0.130460456 | -2.938315513 | 0.000110751 | 0.129847309 | |
| LINC00205:1 | genic | 0.134227412 | -2.897248767 | 0.000170328 | 0.164456594 | |
| lnc-RPL13A-1:8 | unknown | 0.134500098 | -2.894320868 | 3.32E-05 | 0.060463586 | |
| lnc-GGCT-1:52 | unknown | 0.150629783 | -2.73092104 | 9.34E-05 | 0.129847309 | |
| lnc-BLACE-2:3 | unknown | 0.151783277 | -2.719915244 | 0.000309381 | 0.195314876 | |
| RAB30-AS1:32 | intergenic | 0.15405297 | -2.698501596 | 0.000499193 | 0.239664252 | |
| lnc-PPP4C-2:1 | unknown | 0.154503495 | -2.694288625 | 0.000525644 | 0.239664252 | |
| LINC02422:15 | intergenic | 6.157091198 | 2.622248938 | 0.000109617 | 0.129847309 | |
| lnc-TMEM62-2:1 | intergenic | 6.186095391 | 2.62902908 | 0.000604167 | 0.248511527 | |
| lnc-EPHA6-1:5 | unknown | 6.375336742 | 2.672501546 | 0.000442883 | 0.227171112 | |
| lnc-DRICH1-2:4 | unknown | 6.530282447 | 2.707145392 | 0.000264323 | 0.191056782 | |
| lnc-STK26-5:1 | unknown | 7.455048977 | 2.89821783 | 0.000268799 | 0.191056782 | |
| FAM212B-AS1:11 | unknown | 9.570196365 | 3.258548527 | 2.70E-05 | 0.055489627 | |
| lnc-POU5F1B-3:14 | unknown | 11.81373497 | 3.562393246 | 1.05E-06 | 0.004311092 | |
| TCONS_00039064 | intergenic | 14.16593146 | 3.824353562 | 7.54E-07 | 0.004124 | |
| lnc-PIGN-7:4 | unknown | 14.2151 | 3.829352342 | 1.19E-07 | 0.000979498 | |
| lnc-KBTBD4-4:1 | unknown | 27.13128783 | 4.761885624 | 2.03E-12 | 3.33E-08 | |
| Case1_before vs Case2_before | | | | | | |
| lncRNA_id | type | Fold change | log_2_(Fold change) | p value | Regulation | |
| LINC00189:9 | intergenic | 0.003180954 | -8.296324571 | 0.02517845 | Down | |
| lnc-BRWD1-1:4 | unknown | 0.004778446 | -7.709242621 | 0.013207916 | Down | |
| lnc-SETD7-3:21 | unknown | 0.005989854 | -7.383263355 | 0.000124032 | Down | |
| lnc-GABARAPL2-1:2 | unknown | 0.007107396 | -7.13646313 | 1.89E-05 | Down | |
| TUG1:33 | intergenic | 0.007668471 | -7.026845376 | 0.01020696 | Down | |
| lnc-BRF1-13:1 | unknown | 0.010905367 | -6.518817912 | 0.003453113 | Down | |
| lnc-PLEKHM1-1:1 | unknown | 0.010960543 | -6.511536943 | 0.001297304 | Down | |
| lnc-MSH3-2:1 | genic | 0.010989071 | -6.507786822 | 0.014190235 | Down | |
| lnc-AGO3-3:2 | unknown | 0.011887744 | -6.394381263 | 0.011315167 | Down | |
| TCONS_00002040 | genic | 0.011905036 | -6.392284243 | 0.018264819 | Down | |
| lnc-STARD10-1:3 | unknown | 89.33704932 | 6.4811867 | 0.030685021 | Up | |
| lnc-OCIAD2-8:5 | unknown | 94.26718235 | 6.558683701 | 0.002249167 | Up | |
| lnc-CWC15-1:2 | unknown | 106.7877145 | 6.73860187 | 2.74E-06 | Up | |
| lnc-SLC36A1-5:1 | unknown | 114.0411688 | 6.83341092 | 0.026070059 | Up | |
| TP53TG1:1 | intergenic | 130.0778029 | 7.023230985 | 0.00120922 | Up | |
| lnc-PTS-1:19 | unknown | 154.9388155 | 7.275554805 | 8.73E-05 | Up | |
| PRKCQ-AS1:6 | genic | 202.0926973 | 7.65887338 | 0.00041405 | Up | |
| lnc-SMG1-3:1 | unknown | 227.5905656 | 7.830296944 | 0.001392009 | Up | |
| lnc-CMAS-6:1 | unknown | 412.9738813 | 8.68990673 | 0.033011204 | Up | |
| lnc-SYAP1-2:1 | unknown | 553.0142716 | 9.111172902 | 2.14E-05 | Up | |

**Supplymentary Table 4. Detailed information of the top 10 upregulated and 10 downregulated circRNAs.**

| Case1_before vs Case1_after | | | | | |
| --- | --- | --- | --- | --- | --- |
| circRNA_id | Type | Fold change | log_2_(Fold change) | p value | Regulation |
| circRNA_14708\|Chr17:42022399_42022640_+ | exonic | 0.234225 | -2.094033 | 0.0037268 | Down |
| circRNA_24431\|Chr3:167720062_167725473_- | sense-overlapping | 0.2777679 | -1.848048 | 0.0085297 | Down |
| circRNA_27120\|Chr5:61472681_61494410_+ | sense-overlapping | 0.2906954 | -1.78242 | 0.0126354 | Down |
| circRNA_03269\|Chr1:233090061_233095863_- | sense-overlapping | 0.2920744 | -1.775592 | 0.0129597 | Down |
| circRNA_22716\|Chr3:17622648_17625224_- | sense-overlapping | 0.2958712 | -1.756959 | 0.0152681 | Down |
| circRNA_19664\|Chr2:159743061_159748903_+ | sense-overlapping | 0.304 | -1.717857 | 0.0158764 | Down |
| circRNA_10765\|Chr14:89161701_89180806_- | sense-overlapping | 0.3071302 | -1.703078 | 0.0187411 | Down |
| circRNA_25498\|Chr4:48369849_48379848_+ | sense-overlapping | 0.3169361 | -1.657736 | 0.0223554 | Down |
| circRNA_13861\|Chr16:89305206_89317075_- | sense-overlapping | 0.3219377 | -1.635147 | 0.0213492 | Down |
| circRNA_15967\|Chr18:23515856_23516423_+ | sense-overlapping | 0.3258375 | -1.617775 | 0.0258602 | Down |
| circRNA_00520\|Chr1:26729651_26732792_+ | sense-overlapping | 2.635605 | 1.3981342 | 0.0221275 | Up |
| circRNA_15396\|Chr17:68114422_68125480_+ | intergenic | 2.7743345 | 1.4721417 | 0.0424418 | Up |
| circRNA_00461\|Chr1:24531218_24533254_+ | sense-overlapping | 2.7996799 | 1.4852619 | 0.0354173 | Up |
| circRNA_19379\|Chr2:127993187_127996491_- | sense-overlapping | 2.8547798 | 1.5133795 | 0.0336521 | Up |
| circRNA_19681\|Chr2:161179614_161224746_+ | sense-overlapping | 2.9721116 | 1.5714883 | 0.0302506 | Up |
| circRNA_34641\|Chr9:96774788_96776245_- | sense-overlapping | 2.9937556 | 1.5819565 | 0.0281022 | Up |
| circRNA_06932\|Chr12:894562_897681_+ | sense-overlapping | 3.0499135 | 1.6087683 | 0.0222497 | Up |
| circRNA_03395\|Chr1:236824119_236826896_+ | sense-overlapping | 3.344382 | 1.7417396 | 0.0161635 | Up |
| circRNA_32059\|Chr7:140046884_140058034_- | sense-overlapping | 3.6986613 | 1.8870032 | 0.0074087 | Up |
| circRNA_20093\|Chr2:197069830_197090032_- | sense-overlapping | 4.7670985 | 2.2531114 | 0.0016536 | Up |

| Control_before vs Control_after | | | | | |
| --- | --- | --- | --- | --- | --- |
| circRNA_id | Type | Fold change | log_2_(Fold change) | p value | Regulation |
| circRNA_16414\|Chr18:79096476_79176907_+ | sense-overlapping | 0.371141 | -1.42996 | 0.010423 | Down |
| circRNA_08373\|Chr12:110455586_110457634_- | sense-overlapping | 0.382239 | -1.38745 | 0.008844 | Down |
| circRNA_30354\|Chr6:158282263_158314268_+ | sense-overlapping | 0.396219 | -1.33563 | 0.002105 | Down |
| circRNA_06408\|Chr11:86007542_86026367_- | sense-overlapping | 0.399673 | -1.32311 | 0.017695 | Down |
| circRNA_33576\|Chr8:130214556_130236994_- | sense-overlapping | 0.416709 | -1.26289 | 0.02303 | Down |
| circRNA_21369\|Chr20:48688657_48691096_- | sense-overlapping | 0.433258 | -1.2067 | 0.027243 | Down |
| circRNA_03749\|Chr10:12230948_12238485_+ | sense-overlapping | 0.435266 | -1.20003 | 0.026022 | Down |
| circRNA_02009\|Chr1:150226608_150229237_- | sense-overlapping | 0.435657 | -1.19874 | 0.031587 | Down |
| circRNA_00873\|Chr1:39853970_39857417_- | sense-overlapping | 0.441125 | -1.18074 | 0.032685 | Down |
| circRNA_18844\|Chr2:71426460_71427414_+ | exonic | 0.442821 | -1.1752 | 0.034354 | Down |
| circRNA_08872\|Chr13:21161790_21172681_- | sense-overlapping | 2.161055 | 1.111736 | 0.036347 | Up |
| circRNA_30030\|Chr6:135192324_135203324_+ | sense-overlapping | 2.208005 | 1.142743 | 0.03136 | Up |
| circRNA_21045\|Chr20:34029769_34031604_+ | sense-overlapping | 2.224066 | 1.1532 | 0.027363 | Up |
| circRNA_20256\|Chr2:202555252_202556531_+ | exonic | 2.322063 | 1.215407 | 0.028894 | Up |
| circRNA_28425\|Chr5:169747396_169761625_+ | sense-overlapping | 2.359632 | 1.238562 | 0.017916 | Up |
| circRNA_11946\|Chr15:64112573_64118854_+ | sense-overlapping | 2.367563 | 1.243403 | 0.016209 | Up |
| circRNA_22559\|Chr3:9669432_9671170_+ | sense-overlapping | 2.415124 | 1.272097 | 0.021013 | Up |
| circRNA_18852\|Chr2:71526220_71539239_+ | sense-overlapping | 2.487742 | 1.314837 | 0.008379 | Up |
| circRNA_05667\|Chr11:33105567_33106064_- | sense-overlapping | 2.812123 | 1.49166 | 0.004872 | Up |
| circRNA_00389\|Chr1:21221747_21228041_- | sense-overlapping | 3.254953 | 1.702637 | 0.001951 | Up |

| Case1_before vs Case2_before | | | | | |
| --- | --- | --- | --- | --- | --- |
| circRNA_id | Type | Fold change | log_2_(Fold change) | p value | Regulation |
| circRNA_13799\|Chr16:84979189_84981994_- | sense-overlapping | 0.058927 | -4.08492 | 0.000809 | Down |
| circRNA_31503\|Chr7:92225720_92234923_- | sense-overlapping | 0.06515 | -3.94009 | 0.006568 | Down |
| circRNA_29691\|Chr6:100715462_100725703_- | sense-overlapping | 0.071835 | -3.79917 | 0.032708 | Down |
| circRNA_35644\|ChrX:65051462_65113813_+ | sense-overlapping | 0.073611 | -3.76393 | 0.049872 | Down |
| circRNA_22268\|Chr22:38493710_38501280_- | sense-overlapping | 0.074258 | -3.75131 | 0.029214 | Down |
| circRNA_27011\|Chr5:54154580_54171928_- | sense-overlapping | 0.076332 | -3.71156 | 0.039023 | Down |
| circRNA_08027\|Chr12:89609937_89635251_- | sense-overlapping | 0.078482 | -3.6715 | 0.04374 | Down |
| circRNA_02322\|Chr1:161102048_161102375_- | sense-overlapping | 0.082957 | -3.5915 | 0.043582 | Down |
| circRNA_29771\|Chr6:108928088_108936999_+ | sense-overlapping | 0.084625 | -3.56277 | 0.049354 | Down |
| circRNA_24355\|Chr3:155910692_155925366_+ | sense-overlapping | 0.091568 | -3.44901 | 0.042037 | Down |
| circRNA_24120\|Chr3:141512163_141529802_+ | sense-overlapping | 15.20225 | 3.926213 | 0.040338 | Up |
| circRNA_20237\|Chr2:201760881_201761818_- | exonic | 15.24466 | 3.930232 | 0.002899 | Up |
| circRNA_33022\|Chr8:70213903_70296800_- | sense-overlapping | 15.92594 | 3.993306 | 0.037846 | Up |
| circRNA_08756\|Chr12:128875914_128888744_+ | sense-overlapping | 16.17309 | 4.015523 | 0.020737 | Up |
| circRNA_10223\|Chr14:54408745_54415930_+ | sense-overlapping | 16.43501 | 4.038701 | 0.002716 | Up |
| circRNA_02336\|Chr1:161778244_161802272_+ | sense-overlapping | 17.30344 | 4.112987 | 0.019241 | Up |
| circRNA_05379\|Chr11:3731391_3735324_- | sense-overlapping | 17.9866 | 4.168851 | 0.023461 | Up |
| circRNA_24731\|Chr3:185614564_185624082_+ | sense-overlapping | 18.36028 | 4.198516 | 0.037736 | Up |
| circRNA_36007\|ChrX:136012958_136016758_+ | sense-overlapping | 19.81447 | 4.308483 | 0.004333 | Up |
| circRNA_30514\|Chr7:3619080_3642105_+ | sense-overlapping | 24.55427 | 4.617902 | 0.000675 | Up |
